# Supplementary material for: A cross-modality enhancement of defensive flight via parvalbumin neurons in zona incerta
Source: eLife. 2019 Apr 15;8:e42728. doi: 10.7554/eLife.42728 (PMC6486150; doi:10.7554/eLife.42728)
Supplement: Supplementary file 1. — Data are presented as mean ± SD. Two-sided paired t-test were performed to compared values between control and manipulation conditions. The type of experiment is shown by the corresponding figure number in main figures. [file elife-42728-supp1.pdf]

	Response Latency			Time to Peak		
	Control (s)	Manipulation (s)	p-value	Control (s)	Manipulation (s)	p-value
Figure 1A-C	0.171±0.049	0.157±0.054	0.604	1.843±0.730	1.814±0.876	0.937
Figure 1D-F	0.400±0.255	0.520±0.683	0.569	1.940±0.428	2.040±0.230	0.528
Figure 2C-E	0.544±0.341	0.600±0.386	0.955	1.900±0.753	2.481±0.964	0.129
Figure 2F-H	0.720±0.192	0.560±0.270	0.327	1.700±0.579	1.920±0.482	0.585
Figure 3A-C	0.443±0.237	0.491±0.221	0.066	2.514±1.320	2.757±1.655	0.716
Figure 3D-F	0.700±0.430	0.740±0.365	0.597	2.940±1.161	2.800±0.656	0.786
Figure 3G-I	0.675±0.362	0.550±0.359	0.499	1.738±0.537	1.388±0.564	0.224
Figure 4C-E	0.800±0.430	0.840±0.261	0.862	2.260±0.796	2.400±0.946	0.505
Figure 4F-H	0.400±0.187	0.700±0.367	0.259	3.760±1.240	2.860±0.602	0.061
Figure 4I-K	0.5000±0.141	0.5200±0.239	0.799	2.5000±0.593	2.2830±0.568	0.734

Supplemental file 1 Analysis of temporal profiles of speed traces in different sets of experiments. Data are presented as mean ± SD. Two-sided paired t-test were performed to compared values between control and manipulation conditions. The type of experiment is shown by the corresponding figure number in main figures.
